# Comparison between Nasopharyngeal and Saliva Samples for the Detection of Respiratory Viruses in Children with Acute Lower Respiratory Tract Infections: A Pilot Study

**DOI:** 10.3390/children10050899

**Published:** 2023-05-19

**Authors:** Danilo Buonsenso, Piero Valentini, Francesco Mariani, Silvia Di Noi, Sofia Mazza, Ivana Palucci, Maurizio Sanguinetti, Michela Sali

**Affiliations:** 1 Department of Woman and Child Health and Public Health, Fondazione Policlinico Universitario A Gemelli IRCCS, 00168 Rome, Italy; piero.valentini@policlinicogemelli.it (P.V.); francesco.mariani.100292@gmail.com (F.M.); dinoisilvia@gmail.com (S.D.N.); 2 Global Health Research Institute, Istituto di Igiene, Università Cattolica del Sacro Cuore, 00168 Rome, Italy; 3 Medicine and Surgery, Università Cattolica del Sacro Cuore, 00168 Rome, Italy; sofia.mazza97@libero.it; 4 Dipartimento di Scienze Biotecnologiche di Base, Cliniche Intensivologiche e Perioperatorie, Sezione di Microbiologia, Università Cattolica del Sacro Cuore, 00168 Rome, Italy; ivana.palucci@policlinicogemelli.it (I.P.); maurizio.sanguinetti@policlinicogemelli.it (M.S.); michela.sali@unicatt.it (M.S.); 5 Dipartimento di Scienze di Laboratorio e Infettivologiche, Fondazione Policlinico Universitario A Gemelli IRCCS, 00168 Rome, Italy

**Keywords:** respiratory viruses, saliva, children, nasopharyngeal, COVID-19

## Abstract

Purpose: During the COVID-19 pandemic, the use of salivary swabs (SS) to detect the SARS-CoV-2 virus has been implemented and widely studied in adults and children. However, the role of SS in detecting other common respiratory viruses in children is poorly investigated. Methods: Children younger than 18 years of age admitted with respiratory signs and symptoms underwent both nasopharyngeal and SS procedures. Sensitivity, specificity, positive predictive value (PPV), and negative predictive value (NPV) of SS were calculated, considering the nasopharyngeal swab result as the gold standard. Results: A total of 83 patients (44 females, 53%) underwent both nasopharyngeal and SS procedures. Overall, the sensitivity of SS was 49.4%. Sensitivity according to different respiratory viruses ranged from 0% to 71.43%, while the specificity ranged from 96% to 100%. Negative predictive value ranged from 68.06% to 98.8%, while positive predictive value ranged from 0 to 100%. SS sensitivity in patients younger than 12 months of age was 39.47%, while in patients older than or equal to 12 months of age it was 57.78%. Patients with negative SS had a significantly lower median age (8.5 months (15.25) vs. 23 months (34), *p* = 0.001) and a significantly lower quantity of median saliva collected for salivary analysis (0 μL (213) vs. 300 μL (100), *p* < 0.001). Conclusions: SS has a relatively low sensitivity in detecting common respiratory viruses in children with LRTI, with a lower probability in younger children (and in particular those younger than 6 months of age) or those from whom we have collected lesser amounts of saliva. New strategies to improve saliva collection are needed for testing on a larger study population.

## 1. Introduction

Lower respiratory tract infections (LRTIs) represent a major cause of childhood morbidity and mortality worldwide [1]. Viruses account for the majority of cases in all age groups, with some of them being particularly relevant and common, such as the respiratory syncytial virus (RSV), bocavirus, rhinovirus, enteroviruses, influenza viruses, and, since 2019, the SARS-CoV-2. These viruses account for several common childhood respiratory diseases, such as bronchiolitis, bronchitis, wheezing, croup, and viral pneumonia [1]. Although these viruses are particularly dangerous for infants younger than one year of age and those with comorbidities (chronic lung diseases and congenital or acquired immune deficiencies) [1], all age groups from newborns to older adolescents, are susceptible.

Historically, these infections are diagnosed with a nasal or nasopharyngeal swab in children with compatible clinical syndromes, since tracheal aspirates or broncho alveolar lavage are not routinely feasible in children, particularly in those not requiring mechanical ventilation [2]. Although a positive nasopharyngeal test cannot definitely differentiate colonization from disease and cannot be automatically translated to the presence of the virus in the lower respiratory tract, there is general agreement that the presence of a virus in the upper respiratory tract can be interpreted as a causative agent when the clinical picture is coherent [2].

In addition, during the COVID-19 pandemic, nasopharyngeal swabs were and still are the gold standard collection technique for the diagnosis of SARS-CoV-2 infection via RealTime PCR (RT-PCR) [3,4]. However, during the pandemic, the need for performing multiple tests even on the same patient, and particularly on children, as well as the need for rapid tests to be used as screening in particular settings such as schools, has led to the need for investigating alternative routes to diagnose COVID-19 [5,6]. In this setting, saliva has been tested by several groups in both adult and pediatric patients as an alternative route to diagnose SARS-CoV-2 infection and has been found to be a valid and accurate alternative in several settings [4].

However, children have been less involved in SARS-CoV-2 compared to adults, and, as soon as the restrictive measures were relaxed, a rebound of traditional respiratory viruses was documented worldwide, even in uncommon “out-of-season” periods compared to pre-pandemic seasonalities [7]. In such a context, a more comfortable route to investigate not only the presence of SARS-CoV-2 but also other more common pediatric viruses (above all, RSV, but also bocavirus, enterovirus, rhinovirus, etc.) may be particularly useful. Nevertheless, to our knowledge, the accuracy of saliva for the detection of common childhood respiratory viruses has never been addressed so far. For this reason, we performed this pilot prospective study in order to evaluate the sensitivity of saliva samples (SSs) compared to the NPS swabs in children admitted to our Institution with signs and symptoms of lower respiratory tract infection.

## 2. Materials and Methods

### 2.1. Study Design

This is a prospective observational pilot cohort study.

### 2.2. Population

We included pediatric patients younger than 18 years of age admitted with signs and/or symptoms of acute respiratory illness (dyspnea, wheezing on auscultation, rales or crackles on auscultation, acute respiratory distress syndrome, clinical diagnosis of bronchiolitis according to the Italian guidelines, radiological diagnosis of pneumonia, fever) recruited in the period from 1 September 2021 to 31 March 2022. The legal guardians of all study participants provided written informed consent. The study was approved by the Ethic Committee of the Gemelli University Hospital of Rome (ID 4990, prot n 0020257/22).

We included in this study only children admitted with respiratory illnesses whose guardians approved participation. Patients were excluded if guardians did not provide consent to collect saliva samples in addition to nasopharyngeal swabs, saliva samples were collected >12 h after the nasopharyngeal, or patients had symptoms suggesting infectious illnesses other than respiratory (e.g., gastroenteritis, osteomyelitis).

The primary aim of this study was to compare the saliva swab with the routine nasopharyngeal swab (NSP) in the detection of the most common respiratory viruses in children admitted to our Institution for LRTIs.

The secondary objective was to assess whether clinical and demographic data may affect the results of these two examination methods.

### 2.3. Respiratory Specimen Collection

In our institution, the nasopharyngeal swab represents the accepted method for diagnosing viral respiratory infections and is now mandatory for every admitted child to exclude SARS-CoV-2 infection. In children with respiratory symptoms, the nasopharyngeal swab is also used for other respiratory viruses.

**Figure 1:**
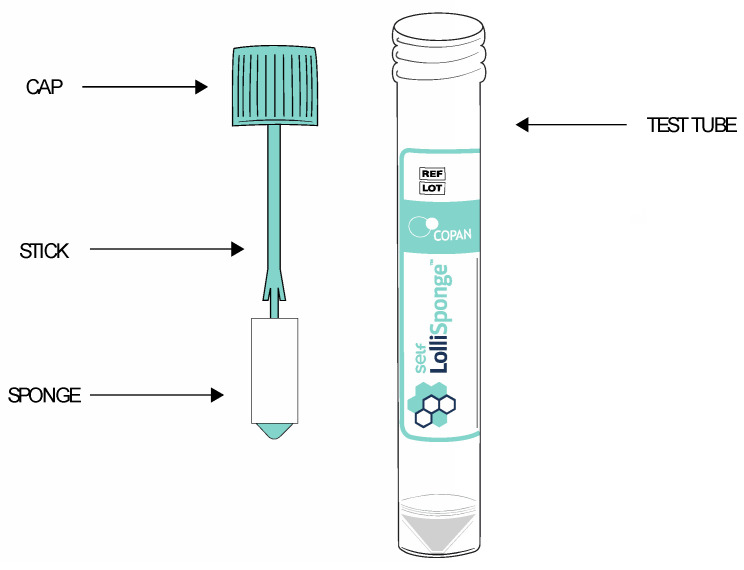
Copan LolliSponge™ (Self LolliSponge™ instructions for use COPAN) (https://www.copangroup.com/product-ranges/lollisponge/, accessed on 1 May 2023).

The nasopharyngeal swab and saliva were collected by the symptomatic children at the time of admission. All samples were collected by a specifically trained 4th year resident in pediatrics in order to avoid bias in sample collection. Nasopharyngeal samples were collected using a viral transport medium by means of two swabs: one for the nose (to be performed in both nostrils) and one for a pharyngeal swab. The saliva samples were collected about one hour after the nasopharyngeal swab with a dedicated salivette (Lollisponge salivette) (Figure 1). In depth, saliva samples were collected as suggested by the manufacturer. Briefly, the swabs were frictioned on the tongue and on the internal side of both cheeks close to one minute under chronometer control. Both NPS and saliva samples were immediately transported to the microbiology unit of our institute, kept refrigerated at 4 °C, and processed within 8–12 h of collection.

In depth, for all children, the following data were collected:

- Age (months), sex, and ethnicity;
- NSP, date, and result;
- Salivary swab, date, and result;
- Amount of saliva collected;
- Comorbidities;
- Asymptomatic/symptomatic;
- Presence of fever, cough, or respiratory distress;
- Need of oxygen support or not;

Following specimen collection, the device was centrifuged in order to extract the saliva from the sponge. The recommended protocol is 450 G for 60 s. Using a micropipette, remix the saliva specimen that has been deposited on the bottom of the test tube in order to homogenize it to dissolve any pellets that have formed due to centrifugation and aliquot it into dedicated extraction test tubes [8].

### 2.4. Pathogen Analysis

Pathogen detection was performed using QIAstat-Dx Respiratory SARS-CoV-2 Panel (Qiagen, Hilden, Germany), the ePlex^®^ Respiratory Pathogen Panel (ePlex RPP) by GenMark Diagnostics, Inc. (Carlsbad, CA, USA), and the Aptima SARS-CoV-2 assay on the Panther instrument (Hologic, Inc., San Diego, CA, USA).

The ePlex RPP assay as used in this study is able to detect 25 respiratory pathogens, including the differentiation of subtypes of influenza A virus, parainfluenza virus, and respiratory syncytial virus (RSV). ePlex RPP assay technology is based on the principles of competitive DNA hybridization and electrochemical detection, which are highly specific and have sensitivities comparable to other molecular systems [9]. According to the manufacturer’s instructions, 200 μL of the respiratory sample was pipetted into a buffer tube (supplied by the manufacturer) and, after vortex mixing, transferred into the ePlex RPP test cartridge. After approximately 90 min, the results of the analysis of the pathogen targets were reported as positive or not detected.

The QIAstat-Dx RP assay is a RealTime multiplexed PCR test. The platform consists of automated nucleic acid extraction, reverse transcription, PCR, and fluorescence detection. The platform consists of automated nucleic acid extraction, reverse transcription, PCR, and fluorescence detection. The test is intended for the qualitative detection and simultaneous identification of multiple viral and bacterial respiratory nucleic acids in nasopharyngeal swabs. The test was performed as described in the manufacturer’s instructions and allows the simultaneous and rapid (~70 min) detection of 18 viral pathogens, including SARS-CoV-2, and 3 bacterial pathogens (Mycoplasma pneumoniae, Legionella pneumophila, and Bordetella pertussis) that cause respiratory infections [10,11]. Brifley: A total of 300 μL of the respiratory sample was transferred into a QIAstat-Dx RP test cartridge. The barcode of the test cartridge and the barcode of the corresponding sample were scanned by the QIAstat-Dx operational module, followed by the loading of the test cartridge into the QIAstat-Dx analyzer module and starting the run.

The Aptima SARS-CoV-2 assay combines the technologies of target capture, transcription-mediated amplification (TMA), and dual kinetic assay (DKA), targeting two parts of the ORF1 ab region of the SARS-CoV-2 genome and one internal control [12]. This test is based on end-point transcription-mediated amplification (EP-TMA), which is a binary test for the presence or absence of SARS-CoV-2 [13]. After amplification, chemiluminescent probes hybridize to amplicons and emit light measured by a luminometer in relative light units (RLUs). The Aptima™ SARS-CoV-2 assay was performed following the manufacturer’s instructions. Virus transport medium (500 μL) was manually placed in the Panther™ tube containing 710 μL of lysis buffer. The instrument used 360 μL of this mix for the lysis and capture of nucleic acids.

### 2.5. Statistical Analysis Plan

#### 2.5.1. Sample Size Calculation

To our knowledge, the accuracy of saliva for the detection of common childhood respiratory viruses has never been addressed so far. Given the lack of evidence in the literature, this is a pilot study. As such, no formal sample size calculation is needed. Based on rules of thumb from internal pilot studies, the minimum sample size required would be 20 subjects [14]. However, based on the children commonly observed in our unit, we planned to include 50 or more children who fulfilled the inclusion criteria.

#### 2.5.2. Statistical Analysis

All variables included in the study were first analyzed using descriptive statistics techniques. In depth, qualitative variables were described as absolute and percentage frequencies. The Kolmogorov-Smirnov test was used to assess the distribution of quantitative variables. Data were then expressed either as mean and standard deviation (SD), whether normally distributed, or as median and interquartile range (IQR), otherwise. The agreement between the two diagnostic methods results was assessed by the Chi-square test or Fisher’s exact test for categorical variables and the Mann-Whitney U test for continuous variables if they were not normally distributed.

Sensitivity, specificity, positive predictive value (PPV), and negative predictive value (NPV) were further computed, considering the NSP result as the gold standard, through Excel 2 × 2 tables. In cases where the amount of saliva collected was insufficient for microbiological tests, the result was considered “negative” to reflect real-life applicability.

The same analyses were performed stratified by age (<12 months vs. older and <6 months vs. older).

In addition, we aim to assess whether children’ demographic and clinical data would affect the results of the two types of swabs. A *p*-value <0.05 was considered statistically significant. All analyses were performed using IBM SPSS Statistics 23.0 software (IBM Corporation, Armonk, NY, USA).

All graphs were run using GraphPad Prism Version 9.3.1. software (350).

## 3. Results

### 3.1. Study Population

A total of 83 patients (including 44 females, 53%) performed both tests, NSP collection and salivary swab (SS). The median age at the time of swab was 12 months (IQR 30, min 0 max 204), and 17 patients (20.5%) had comorbidities (Table 1).

At the time of swab collection, 59 patients (71.1%) were pyretic, 65 had a cough (78.3%), 51 (61.4%) had respiratory distress, 3 (3.6%) had rhinitis, and 10 had gastrointestinal disorders (12%). Among the comorbidities, the most represented were genetic syndromes in 6 patients (7.2%) and epilepsy in 4 cases (4.8%). During hospitalization, 48 patients (57.8%) received oxygen therapy.

### 3.2. Comparison of Molecolar Results between Nasopharyngeal Swabs and Sputum Specimens

**Figure 2:**
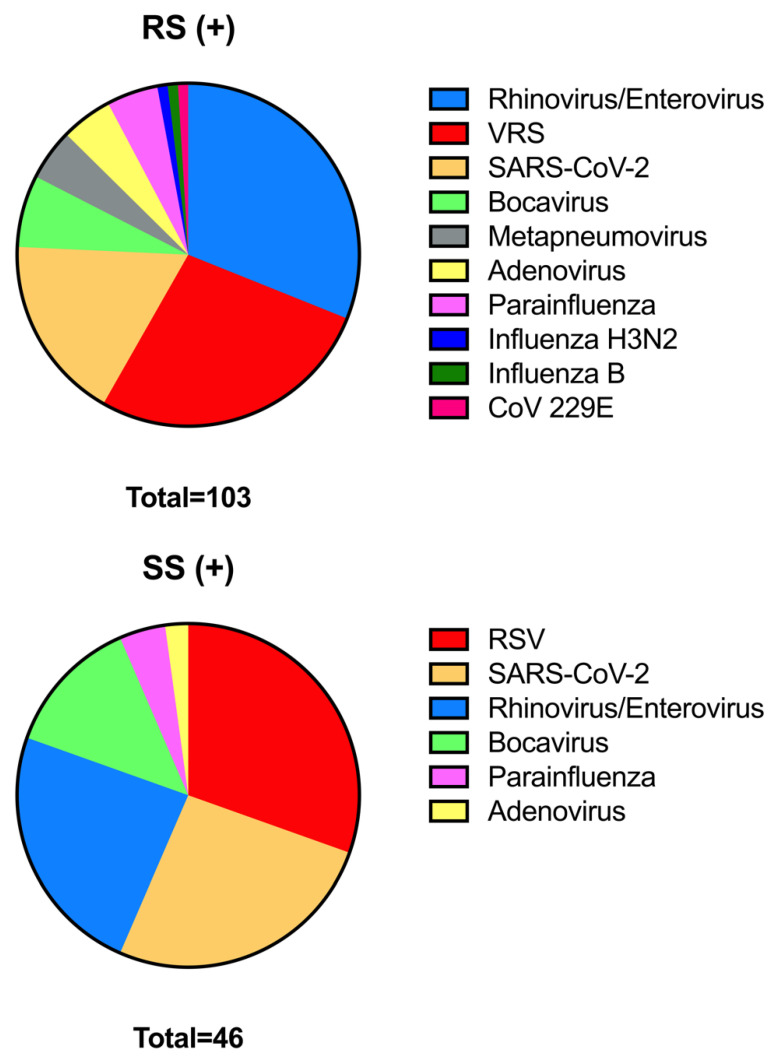
Pie chart representing viruses identified in NSP positive (+) swabs. A total of 103 identifies the number of times a virus was detected in the individual swab; considering that more than one virus was detected in 17 NSP, the total number of positive NSP is 83. Pie chart representing viruses identified in positive (+) salivary swabs (SS). A total of 46 identifies the number of times a virus was detected in a single swab; considering that more than one virus was detected in 5 salivary swabs, the total number of positive salivary swabs is 41.

All patients had a positive NSP swab, and 41 of them (49.4%) also tested positive for a salivary swab, while in 28 cases the amount of saliva collected was insufficient for microbiological tests and therefore considered “negative” to reflect real-life applicability. The results of both swabs according to each viral isolate are shown in Figure 2 and Table 2. For the salivary swab in general, which does not refer to a single virus, in consideration of the absence of patients with a negative NSP, it was possible to evaluate only the sensitivity that resulted in 49.4%.

Table 3 shows the different values of sensibility, specificity, positive predictive value, and negative predictive value in the detection of the different viruses on the salivary swab. The values of sensitivity showed a wide range, passing from 0% to 71.43%, while the specificity was usually higher, passing from a minimum of 96% to a maximum of 100%. Negative predictive value ranged from 68.06% to 98.8%, while, for those cases where it was possible to calculate it, positive predictive value ranged from 0 to 100%. Salivary swab sensitivity in patients younger than 12 months of age was 39.47%, while in patients older than or equal to 12 months of age, it was 57.78%.

Table 4 compares patients with a positive salivary swab and those with a negative salivary swab. Patients with a negative swab had a significantly lower median age (8.5 months (15.25) vs. 23 months (34), *p* = 0.001) and a significantly lower quantity of median saliva collected for salivary analysis (0 μL (213) vs. 300 μL (100) *p* < 0.001).

The relationship between two dichotomous nominal variables (positive/negative) via the contingency table are reported in Table 5.

## 4. Discussion

RealTime polymerase chain reaction (RT-PCR) performed on nasopharyngeal specimens (NPS) is so far the gold standard for the detection of respiratory viruses in patients with lower respiratory tract infections (LTRI). However, nasopharyngeal swabbing has several disadvantages: it requires performance by specifically trained and qualified health care workers and the use of personal protective equipment (PPE) in order to minimize the risk of patient-to-worker transmission; in addition, it is often uncomfortable for the patient, especially for children who are frequently reluctant to perform it. For this reason, saliva may be an alternative sample type, being theoretically less invasive and more easily performed directly by the parent of the patient or by the child himself/herself when he/she is older. To our knowledge, this is the first study to evaluate the diagnostic accuracy of saliva samples in multiplex RealTime PCR testing for the detection of multiple common respiratory viruses in those requiring pediatric care.

Overall, the sensitivity of the salivary swab was 49.40%, with a wide variability in detection rates according to the different isolated viruses. In fact, when analyzing salivary swab sensitivity for each individual virus, the highest sensitivity was recorded for Bocavirus (71.43%) and SARS-CoV-2 (66.67%). Intermediate sensitivity was recorded for RSV (46.43%) and Parainfluenza (40%). In contrast, the lowest sensitivity was recorded for Rhinovirus/Enterovirus (28.13%). For the remaining viruses not detected in salivary swabs, the sensitivity was 0%. The latter group also included Adenovirus, although the salivary swab detected 1 case of positivity compared to 5 positive NSP swabs.

For Bocavirus, the concordance between the two samples—NSP and salivary—was high, having detected HBoV in 7 (8.4%) and 6 (7.2%) cases, respectively, in the study population, with a VPP of the salivary swab of 92.86%. To date, the pathogenesis of Bocavirus is poorly understood due to the lack of experimental animal models or specific cell lines for viral culture; however, it has been commonly detected in the respiratory tract [15]. Although the existence of a selective tropism of the virus for a specific site is not yet known with certainty, the high sensitivity of the salivary swab for HBoV could be related to the high viral load in saliva as a consequence of virion shedding into the oral cavity from the infected respiratory epithelium [16].

A total of 14.5% (12) of the salivary swabs were positive for SARS-CoV-2, compared with a positivity of 21.7% (18) in NSP swabs. The good performance of salivary samples in detecting SARS-CoV-2 could be partly related to the local replication of the virus, as ACE2—the main receptor of the virus in question—is also expressed in the oral mucosa [17] and salivary glands [18], and partly related to the mixing of upper and lower respiratory fluids in saliva, which would, therefore, show a detectable viral load [6].

Several recent studies have evaluated saliva as an alternative sample for SARS-CoV-2 detection [19,20,21,22,23,24,25,26,27,28]. All agree that there is enough viral load present in saliva for detection to be possible; however, comparisons between nasopharyngeal swabs and salivary swabs have led to different conclusions: some studies [22,27,28] have shown lower sensitivity of saliva than the sensitivity of a nasopharyngeal swab, while others [24,26] have found better diagnostic performance of saliva. However, the overall sensitivity of saliva remains debated, and there are several factors that may influence it, so further studies are needed to validate this hypothesis.

Although there are a growing number of studies evaluating the diagnostic accuracy of saliva as an alternative sample for SARS-CoV-2 detection in adults, there is a dearth of information for the pediatric populations. Only a few studies [29,30,31,32] have included children in their populations. Contrary to the general agreement, two studies [33,34] have already suggested that saliva may not be an ideal sample for the diagnosis of COVID-19 in those requiring pediatric care; nevertheless, it is difficult to generalize their result due to a number of limitations, including the small size of the test population.

The low yield of salivary samples in detecting Rhinovirus/Enterovirus could be related to the lower concentration of them in saliva, the nasal mucosa being the selective replication site of RV, as confirmed, in part, by the improved growth of the microorganism at temperatures of 33–35 °C [35]. Because the tropism of Rhinovirus is different from that of other respiratory viruses, the use of saliva in this context should be confirmed in future studies.

The detection rate of Adenovirus from saliva samples was lower than from nasopharyngeal/nasal samples. This could be related to the fact that the primary replication site of Adenoviruses is the non-ciliated respiratory epithelium of the oropharynx [36]. Contrary to what was observed in this study, a previous study [37] reported a significantly higher detection rate of Adenoviruses from saliva samples than from NPS samples.

The utility of saliva as an alternative sample for the detection of respiratory viruses beyond SARS-CoV-2 has been considered in a limited number of studies. Some of the studies [38,39,40] have shown that saliva can be used as a biological material for influenza virus detection, demonstrating a high degree of agreement between the results of nasopharyngeal and salivary swabs. In a previous study [37] that compared nasopharyngeal and salivary samples for the detection of sixteen respiratory viruses, salivary samples showed an overall performance equivalent to that of NPS samples. In another study [41] that, however, included only hospitalized adult patients with severe illness, saliva samples showed high sensitivity and specificity in detecting respiratory viruses (influenza A, influenza B, and RSV).

However, to our knowledge, only one study from 2008 [42] compared pharyngeal and salivary swabs with NPS samples for the detection of four respiratory viruses (RSV, Influenza A, Influenza B, and Parainfluenza) in the pediatric population (age ≤ 17 years) with presumed lower respiratory tract infection. If the NPS sample had detected respiratory viruses by direct fluorescent antigen detection (DFA) or nucleic acid amplification test (NAAT), a pharyngeal and salivary swab analyzed by NAAT was performed for the same virus. The yield of the test on the salivary sample was 74%, which according to the starting hypothesis of the study, is not sufficient to place an LTRI diagnosis.

Aiming to better investigate possible reasons for a negative saliva sample, we compared saliva results according to clinical presentation and age groups. No statistically significant difference was observed between saliva swab-positive and negative subjects with regard to symptoms and comorbidities presented, so they should not affect the test result. Further clinical data collected concerned the need for respiratory assistance; in fact, since these were children with LTRIs, oxygen therapy was set up in 48 cases in order to counter respiratory distress and maintain SatO2 at adequate levels. Therefore, we hypothesized that ongoing respiratory distress or non-invasive oxygen support might have affected saliva production and, therefore, the outcome of salivary swabbing. However, the comparison between patients with positive salivary swabs on oxygen therapy (24; 58.5%) and patients with negative salivary swabs on oxygen therapy (24; 57.1%) was not statistically significant.

In addition, age and the amount of saliva collected influenced the salivary swab result. In subjects with a positive salivary swab, the median age was 23 months (24 IQR), while in subjects with a negative salivary swab, the median age was 8.5 months (15.25 IQR) (*p*-value < 0.001). Nevertheless, by setting 12 months as the cut-off, no statistically significant difference was observed between the two groups. In contrast, when setting 6 months as the cut-off, a statistically significant difference was observed (*p*-value 0.006). As for the amount of saliva collected, in patients with a positive salivary swab, it was 300 μL (100 IQR), while in the patients with a negative salivary swab, it was 0 μL (213 IQR) (*p*-value < 0.001). Therefore, these two data points, the age of the patient and the amount of sample collected, seem to be major factors in saliva sensitivity. Thus, it is possible to hypothesize that in younger children or in those producing less saliva, the diagnosis by salivary swab might be unsuitable (in the latter case because of the risk of having an insufficient sample for laboratory analysis), or an alternative method to collect saliva may be required.

Regarding the age of the patient, several factors could have influenced the result of the salivary swab. For example, since saliva production is partly related to food digestion and the secretory stimulus is mediated by mechanoreceptors present on the walls of the oral cavity and thus by chewing [43], in the first three months of life infants produce only a minimal amount of saliva since their only diet is breast or cow’s milk. Later, as the salivary glands grow and mature as the diet changes and teeth erupt, more saliva is produced [44,45,46]. In addition, it is possible that salivary swabbing in this age group may have been more difficult due to the possible discrepancy between the size of the swab itself and the child’s oral cavity, so it may have been difficult to collect saliva. Finally, the poor compliance of the younger child should not be overlooked, which may have affected the collection of the sample by the operator. Therefore, in this younger population, the use of saliva should be further explored, including new methods of saliva collection. In this study, we have considered an insufficient amount of collected saliva as a negative test. Although from a statistically and microbiological perspective, this may not be fully correct, from a clinical perspective, it reflects a real-world scenario. In routine practice, clinicians need to be sure that a collected sample will provide a result (either negative or positive), and an “inadequate” sample would be an uncomfortable result, specifically for discharged children or those that await results to define the best type of hospital isolation. Therefore, having a positive NPS and an “inadequate/insufficient” saliva swab is a relevant result in terms of routine clinical practice.

This study had limitations to address. First, all included patients had a positive NSP, so it was not possible to directly compare the two samples. As a pilot study, we decided to test children with signs and symptoms of LRTI. Future studies should evaluate larger populations and also include NPS-negative subjects in order to be able to evaluate the diagnostic accuracy (or efficiency) of the salivary swab through the ROC (receiver operating characteristic) curve, particularly through the AUC (area under the curve). Second, quantitative concordance between the two diagnostic methods could not be calculated in this study because different RT-PCR panels that use different “values” to determine the number of cycles required for a sample to amplify and pass a threshold (cut-off) to be considered positive, specifically Ct (cycle threshold) and RLU (relative light unit), were used for viral load determination in swabs of SARS-CoV-2 and other viruses, respectively. In addition, a similar study would be important to address in adults and elderlies, which we had no access to.

## 5. Conclusions

In conclusion, our study showed that saliva samples have a relatively low sensitivity in detecting common respiratory viruses in children with LRTI, with a lower probability in younger children (and in particular those younger than 6 months of age) or those from collected whom we have collected lesser amounts of saliva. In addition, sensitivities varied according to different viruses, suggesting possible virus-specific differences in replication in saliva and nasal epithelium. However, as most viruses were detected in the saliva, our study also highlights the potential future role of saliva sampling in children with LRTI after the implementation of new strategies to collect saliva in younger children and testing them on a larger study population.

## Figures and Tables

**Table 1:** Demographic characteristics of the children included in the study.

**Number of Patients**	83
Gender (n, %)	
Male	39 (47%)
Female	44 (53%)
Age, months (median, IQR)	12 (30)
Ethnicity (n, %)	
Caucasian	68 (81.9%)
Asian	8 (9.6%)
African	3 (3.6%)
Hispanic	4 (4.8%)
Swab type (n, %)	
Nasopharyngeal	79 (95.2%)
Nasal	4 (4.8%)
Comorbidities (n, %)	17 (20.5%)
Isolation of more than one virus at NSP (n, %)	17 (20.5%)
Isolation of more than one virus at salivar swab (n, %)	5 (6%)
Salivary swab result (n, %)	
Positive	41 (49.4%)
Negative	42 (50.6%)

**Table 2:** Swab outcomes (routine and salivary) in the whole population according to each virus.

	Paired	Samples
		83
	Nasopharingeal swab	Salivary swab
Rhinovirus/Enterovirus		
positive	32 (38.6%)	11 (13.3%)
negative	51 (61.4%)	72 (86.7%)
RSV ^1^		
positive	28 (33.7%)	14 (16.9%)
negative	55 (66.3%)	69 (83.1%)
Bocavirus		
positive	7 (8.4%)	6 (7.2%)
negative	76 (91.6%)	77 (92.8%)
SARS-CoV-2		
positive	18 (21.7%)	12 (14.5%)
negative	65 (78.3%)	71 (85.5%)
Influenza B		
positive	1 (1.2%)	0
negative	82 (98.8%)	83 (100%)
Adenovirus		
positive	5 (6%)	1 (1.2%)
negative	78 (94%)	82 (98.8%)
CoV ^2^ 229E		
positive	1 (1.2%)	0
negative	82 (98.8%)	83 (100%)
Metapneumovirus		
positive	5 (6%)	0
negative	78 (94%)	83 (100%)
Parainfluenza		
positive	5 (6%)	2 (2.4%)
negative	78 (94%)	81 (97.6%)
Influenza H3N2		
positive	1 (1.2%)	0
negative	82 (98.8%)	83 (100%)

^1^ Respiratory Syncytial Virus, ^2^ Coronavirus.

**Table 3:** PPV, NPV, sensitivity, and specificity of salivary swab.

	PPV ^1^	NPV ^2^	Sensitivity	Specificity
Salivary swab			49.40% *	
Rhinovirus/Enterovirus	81.82%	68.06%	28.13%	96.08%
RSV ^3^	92.86%	78.26%	46.43%	98.18%
Bocavirus	83.33%	97.40%	71.43%	98.68%
SARS-CoV-2	100.00%	91.55%	66.67%	100.00%
Influenza B		98.80%	0.00%	100.00%
Adenovirus	0.00%	93.90%	0.00%	98.72%
CoV ^4^ 229E		98.80%	0.00%	100.00%
Metapneumovirus		93.98%	0.00%	100.00%
Parainfluenzae	100.00%	96.30%	40.00%	100.00%
Influenza H3N2		98.80%	0.00%	100.00%

^1^ Positive predictive value, ^2^ Negative predictive value, ^3^ Respiratory Syncytial Virus, ^4^ Coronavirus, * overall sensitivity.

**Table 4:** Comparison of demographic and clinical characteristics: patients with negative salivary swab and patients with positive salivary swab.

	Negative Salivary Swab	Positive Salivary Swab	*p* ^1^
	N = 42	N = 41	
Age (median, IQR)	8.5 (15.25)	23 (34)	0.001
Age < 12 months (n, %)	23 (54.8%)	15 (36.6%)	0.097
Age < 6 months (n, %)	19 (45.2%)	7 (17.1%)	0.006
Gender (n, %)			
Male	20 (47.6%)	19 (46.3%)	
Female	22 (52.4%)	22 (53.7%)	0.9
Days between RS ^2^-SS ^3^			
(median, IQR)	2 (2)	2 (3)	0.68
Amount of saliva collected (microliters)			
(median, IQR)	0 (213)	300 (100)	<0.001
Fever (n, %)	27 (64.3%)	32 (78%)	0.17
Cough (n, %)	30 (71.4%)	35 (85.4%)	0.12
Respiratory distress (n, %)	24 (57.1%)	27 (65.9%)	0.41
Rhinitis (n, %)	2 (4.8%)	1 (2.4%)	1
Comorbidities (n, %)	5 (11.9%)	12 (29.3%)	0.05

^1^ *p* value, ^2^ NSP ^3^ Salivary swab.

**Table 5:** Representation on a 2 × 2 table of the distribution of the study population according to the results of the two sample types.

Sample		Nasopharingeal Swab		
		Positive (+)	Negative (−)	
Salivary swab	Positive (+)	41	0	41
	Negative (−)	42	0	42
		83	0	83

## Data Availability

Dataset available upon request to the corresponding author.
